# Production and composition of *Pleurotus ostreatus* cultivated on Lithovit^®^-Amino25 supplemented spent substrate

**DOI:** 10.1186/s13568-020-01124-1

**Published:** 2020-10-21

**Authors:** Layla Naim, Mohammed A. Alsanad, Nidal Shaban, Zeina El Sebaaly, Sami Abou Fayssal, Youssef N. Sassine

**Affiliations:** 1 University of Forestry, 10 Kliment Ohridski blvd, 1797 Sofia, Bulgaria; 2 Department of Environment and Agricultural Natural Resources, College of Agricultural and Food Sciences, King Faisal University, P.O. Box 420, Al-Ahsa, 31982 Kingdom of Saudi Arabia; 3 Department of Plant Production, Faculty of Agriculture, Lebanese University, Beirut, Lebanon; 4 Department of Agricultural Biotechnology, College of Agricultural and Food Sciences, King Faisal University, P.O. Box 420, Al-Ahsa, 31982 Kingdom of Saudi Arabia

**Keywords:** Supplementation, Fiber fractions, Degradability, Yield, Nutritional value

## Abstract

Supplementation of the spent oyster substrate enhances its nutritional properties to produce a new mushroom cropping cycle. The study investigated the potential of a nano-fertilizer (Lithovit^®^-Amino25) with an admixture of 25% l-amino acids on *Pleurotus ostreatus* production, proteins, and amino acid contents. The product applied at spawning (t1), after the first harvest (t2), and at both timings (t3), in two doses: 3 g/kg (C1) or 5 g/kg (C2). Compared with control (C0t0), the first harvest was earlier by 2.3–3.3 days in C1t1 and C2t1. The biological yield of the second harvest was improved by 28.0% in C2t2. Superior results were in C1t3 where the number of crops increased to four, biological efficiency was optimized (117.3%) at the third harvest, and biological and economic yields increased by 36.7% and 36.4%, respectively. Lignin was the most degraded in C1t3, while residual cellulose, hemicellulose, neutral detergent fiber, and acid detergent fiber were higher in all treated substrates than in control. In C2t1, mushrooms were the richest in proteins, while in C1t1, they were the richest in the essential amino acids threonine, valine, isoleucine, leucine, and histidine. Lithovit^®^-Amino25 has a high potential for use in *P. ostreatus* production.

## Introduction

In the last decade, the acknowledgment that conventional farming technologies would not have the option to build profitability any further has increased nanotechnology’s interest (Mukhopadhyay 2014). Development in agriculture can be accomplished uniquely by increasing productivity through nanotechnology’s effective use (Selva and Balakrishnan 2017). Several research studies have pointed out their effects on improving growth, yield, and quality parameters of crops (Duhan et al. 2017), like tomato (Sajyan et al. 2018, 2019), grapevines (Sabir et al. 2014; Sassine et al. 2019) and many others. By concentrating on the exceptional properties of materials rising out of nanometric size, nanotechnology has the potential to revolutionize in the food sector (Baruah et al. 2008).

Mushrooms are edible fungi of commercial importance (Shivhare et al. 2004), used for therapeutic and nourishment purposes (Wani et al. 2010). Mushrooms of Pleurotus species, commonly known as oyster mushrooms, are globally highly intriguing for production because of their capacity to develop in a wide range of temperatures and use accessible lignocellulosic materials (Stamets 2000; Baysal et al. 2003; Royse 2003). In particular, *Pleurotus ostreatus* (Jacq.). P. Kumm. 1871 is the second-largest commercially cultivated edible mushroom, constituting approximately 27% of the total global production (Royse 2014). On an industrial scale, *P. ostreatus* is grown on cereal straw, mainly wheat straw (Rühl and Kües 2007). However, in many regions of the world, wheat straw is becoming less available and expensive (Masevhe et al. 2015; Picornell-Buendía et al. 2016a).

In mushroom producing regions, the spent mushroom substrate (SMS), which is the growing material left after several mushroom harvests, is generated in large amounts as 1 kg of fresh mushrooms brings about 5 kg of a spent substrate (i.e., 2 kg dry weight) (Finney et al. 2009). SMS are bulky products long considered a waste stream (Pardo-Giménez et al. 2012). The traditional methods of discarding or burning it are neither eco-friendly nor economic (Oei et al. 2007; Carrasco et al. 2018). On the other hand, SMS is highly nutritious as it is composed of lignocellulosic residues and fungal mycelium. Thus, it constitutes an accessible and low-cost substrate for mushroom cultivation (Grimm and Wösten 2018). However, SMS may not produce excellent mushroom yield by itself because of the reduction in nutrients due to their subsequent utilization by mushroom mycelium (Sharma and Jandaik 1985). Recycling of such substrate through amendment with nutritional supplements, especially protein-rich ones, to help further mushroom production is a practical choice to adapt to the high volume of this waste material (Pardo-Giménez et al. 2011; Pardo-Giménez et al. 2012; Picornell-Buendía et al. 2015, 2016a, b).

Commercial nutritional supplements initially developed for use in the cultivation of Agaricus spp. are based on proteins, lipid/protein blends, carboxylic acids, or minerals (Burton et al. 2015). On an experimental scale, amino acids could ameliorate mushroom performance (Sanchez et al. 2002). Still, their use as nutritional additives for *P. ostreatus* is tested only in submerged liquid cultures (Adebayo-Tayo et al. 2011). On a general basis, during mushroom cultivation, the supplement’s choice, and the correct timing and application methods are fundamental for getting the expected outcomes (Desrumaux et al. 1999). Usually, supplements with slow nutrient release formulas are applied at the end of the substrate preparation, to promote vegetative development all through the substrate (Naraian et al. 2009). They are also used at the end of the spawn run to advance the mushroom colonization and improve mushroom fructification (Pardo-Giménez et al. 2016).

Furthermore, nanometric size supplements, specially developed for mushroom cultivation, have not been reported yet. Otherwise, the use of nano-fertilizers, initially developed for use on plant crops as supplements for the growing substrate of *P. ostreatus*, had recently come out with meaningful results, mainly when the product was applied twice during the production cycle (Naim et al. 2020). Consequently, the current study investigated the effect of a nano-fertilizer containing an admixture of 25% l-amino acids, in different doses and application timings on *P. ostreatus* growth, production, and amino acid composition.

## Materials and methods

### Experimental treatments

The effect of Lithovit^®^-Amino25 (assigned as nano-amino), sourced from Tribodyn AG Company, Northelm, Germany, was tested in two separate doses: C1: 3 g/kg, C2: 5 g/kg, and three timings of application: t1: at spawning, t2: after the first harvest, t3: at spawning and after the first harvest. Six experimental treatments: C1t1, C1t2, C2t1, C2t2, C1t3, and C2t3, were arranged in a complete factorial design with two factors (dose and timing of application) and ten replicates (bags) per treatment. Experimental treatments were compared to a non-treated substrate or control (C0t0). Lithovit^®^-Amino25 is a nitrogen fertilizer with a 25% admixture of 16 water-soluble vegetable l-amino acids, suitable for use in organic farming (according to Regulation (EC) No. 834/2007-European Community), and having the following composition: 50.0% calcium carbonate (CaCO_3_), 28.0% calcium oxide (CaO), 9.0% silicon dioxide (SiO_2_), 3.0% total nitrogen (N), 1.8% magnesium oxide (MgO), 0.5% iron (Fe), and 0.02% manganese (Mn). The product is obtained from the addition of highly energized 16 water-soluble l-Amino acids to Lithovit particles, created by tribodynamic activation and micronization of dolomite (Bilal 2010).

### Substrate preparation, spawning and cropping

The spent substrate was procured by a local mushroom farm “Gourmet” after one growing oyster mushroom cycle. After 1 week of sun-drying, the substrate shopped and mixed with wheat straw (1:1, w/w mixture). The resulting mixture was pasteurized using boiling water for 8 h at 60–65 °C and then left to cool down to 25 °C (spawning temperature) (Pardo-Giménez et al. 2012). After that, spawning was done using grain spawn of M2175 strain, imported from Mycelia Company, Deinze, Belgium, at a 5% rate (50 g of spawn per kg of the substrate). Spawned substrates were filled into perforated transparent polyethylene bags of 60 × 40 cm, with holes of 20 mm diameter at their sides. Bags were then incubated in a cropping chamber in dark conditions at 23–25 °C. The room was well-sealed, with climate control facilities. It was continuously moistened to keep relative humidity levels in the range of 80–90%. At the end of spawn run stage (complete substrate colonization), stimulation of fruit body formation was carried out by lighting (200 LUX light source), reduction of the temperature inside the chamber to around 15 °C, and ventilation to lower CO_2_ levels and maintain them below 700 ppm.

### Chemical analysis

At the Lebanese Agricultural Research Institute (LARI)-Tal Amara station, samples of the initial substrate (Table 1) were evaluated for moisture (%), organic matter (%), carbon (%), and nitrogen (%) contents, as well as C/N ratio, and pH. Total carbohydrates (%) (Anthrone method), total protein (%) (Kjeldahl method), crude fiber (%) (AOAC 962.09 standards), and total fat content (AOAC 1984) were evaluated at the “Lebanese Food Drugs and Chemicals Administration (LFDCA)” Lebanese University-Hadath. Furthermore, fiber fractions of residual substrates; cellulose, hemicellulose, lignin, neutral detergent fiber (NDF), acid detergent fiber (ADF), and acid detergent lignin (ADL) were determined on dry samples by ANKOM technology method, filter bag technique (08-16-06, 08 − 05) following AOAC official method of analysis (AOAC 2019a, b). Moreover, the mushroom composition was analyzed using samples of the mushroom pileus. Total protein content was determined using the macro-Kjeldahl method (N × 4.38) (Reis et    al. 2012). Analysis of amino acid composition was performed using the Young Li amino acid analyzer applying fluorescence detection using o-phthalaldehyde (OPA) and post-column derivatization method. The amino acid analysis was performed using native samples of mushrooms which were sampled at first (concerning the treatments C1t1 and C2t1), second (concerning the treatments C1t2 and C2t2), or third (concerning the treatments C1t3 and C2t3) harvest. The different tests were performed in triplicates.

**Table 1:** Properties of the growing substrate (WS + SOS, 1:1)

% dry weight	
Organic matter	82.8
Carbon	48.1
Nitrogen	1.1
Total carbohydrates	30.54
Total protein	7.5
Crude fiber	30.44
Fats	2.17
Cellulose	35.95
Hemicellulose	13.14
Lignin	6.59
Neutral detergent fiber	59.26
Acid detergent fiber	46.11
Acid detergent lignin	10.17
Moisture (%)	15.6
C/N ratio	43:1
pH (1:5)	5.2

### Measurements

The date of spawn run initiation (days after spawning: DAS) indicated the appearance of first white mycelial patches at the inner side of inoculated bags. Before filling the bags with inoculated substrates, squares of 5 × 5 cm were drawn on their sides. The time to half and complete mycelial colonization of the substrate was recorded when half or all squares became white. The surface mycelial density corresponded to the degree of substrate colonization by the mycelium. It was evaluated at the time of complete mycelial colonization by assigning: (1) to poor running growth, (2) to mycelium growing throughout the bag but not uniformly white, and (3) to mycelium growing throughout the bag and uniformly white (Yang et al. 2013). The number of mushroom bunches, the weight of bunches, fruit body number, and fruit body weight were recorded at each harvest. Economic yield corresponded to mushrooms’ total weight after removal of the base of stalks (Girmay et al. 2016). Biological efficiency (BE) was determined per treatment as: total fresh weight of mushrooms (g)/initial dry weight of substrate (g) × 100 (Oseni et al.  2012). At each harvest, ten representative mushrooms of uniform size were sampled from each treatment to evaluate the pileus diameter and length and stipe diameter and length, using a sliding caliper.

### Data analysis

Statistical analysis was carried out using SPSS 25^®^, by applying one-way ANOVA and Duncan’s multiple range tests. Pearson’s correlations and stepwise multiple regression analysis were applied to investigate the relation between the biological yield and mushroom indicators, and test the contribution of each mushroom indicator (as a predictor) to variation of economic yield at each harvest. All tests applied considering a *P*_*value*_ < 0.05.

## Results

### Mycelia growth and first harvest

Assessment of mycelia growth (dates of spawn run initiation, 50% substrate colonization, 100% substrate colonization, and mycelial density), and pinhead initiation (Table 2) proved a non-significant effect of supplementation compared with control. However, the first harvest was earlier by 3.3 and 2.3 d following the application of the respective doses 3 g/kg and 5 g/kg of nano-amino at spawning.

**Table 2:** Mycelia growth and pin head initiation following nano-amino application at spawning

Indicators	Treatments			
	C0t0	C1t1	C2t1	*P*_*value*_
Mycelia density	2.7a	2.3a	2.0a	ns
Days after spawning (DAS)				
Spawn run initiation	2.3a	2.0a	2.0a	ns
50% colonization	7.0a	7.0a	8.0a	ns
100% colonization	8.7a	9.7a	10.0a	ns
Pinhead initiation	34.3a	33.0a	31.0a	ns
First harvest	40.0b	36.7a	37.7a	0.026

Values are means; means within the same row followed by the same letters are not significantly different at *P* < 0.05 according to Duncan’s multiple range test, ns: non-significant effect

### Mushroom production at the first harvest

Supplementation of the growing substrate with nano-amino at spawning has caused a significant reduction in the average fruit body number, bunches weight, and economic yield at the first harvest (Table 3). On the contrary, it caused a significant improvement in the average fruit body weight (by 18.1 and 12.4 g on average), pileus diameter (by 4.5 and 3.4 cm on average), pileus length (by 2.0 and 2.7 cm on average), and the ratio PD/SL (by 0.3 and 0.5) with the respective doses of 3 g/kg and 5 g/kg. The average stipe diameter increased by 0.3 cm with 5 g/kg nano-amino, and the average stipe length by 2 cm with 3 g/kg nano-amino. The first harvest’s biological and economic yields decreased significantly in the treatment C2t1 compared to control (reduction by 22.7% and 21.0% on average).

**Table 3:** Effect of nano-amino application on the first harvest

Indicators	Treatments			
	C0t0	C1t1	C2t1	*P*_*value*_
Bunches number	3.7a	3.3a	2.7a	ns
Fruit body number	20.0c	15.7b	8.7a	0.000
Bunches weight (g)	177.3b	127.0a	135.1a	0.001
Fruit body weight (g)	21.0a	39.1a	33.4a	ns
Pileus diameter (cm)	7.1a	11.6b	10.5b	0.000
Stipe diameter (cm)	1.8ab	1.8a	2.1b	ns
Stipe length (cm)	6.5a	8.5b	6.7a	0.000
Pileus length (cm)	5.9a	7.9b	8.6b	0.000
PD/SL	1.1a	1.4b	1.6c	0.000
Biological yield (g/bag)	429.6b	408.3b	332.2a	0.001
Economic yield (g/bag))	374.9b	325.2a	296.5a	0.004

Values are means; means within the same row followed by the same letters are not significantly different at *P* < 0.05 according to Duncan’s multiple range test, ns: non-significant effect

### Mushroom production at the second harvest

In comparison with control, the average number of bunches at the second harvest (Table 4) increased by 2.0 following 3 g/kg nano-amino application at timing 2 (C1t2). Additionally, the average fruit body number was higher by 11.3 and 6.3 following 5 g/kg nano-amino application at the respective timings 2 and 3. Besides, a significant improvement was recorded in the bunches’ weight of C2t2 (by 58.5 g on average), and fruit body weight (by 10.5 g on average) of C1t3. Pileus diameter and pileus length increased in the treatments C1t1 (by 1.8 and 0.9 cm on average) and C1t3 (by 2.2 and 0.8 cm). On the other hand, both tested doses applied at timing 2 caused a significant average length reduction. Also, 3 g/kg nano-amino application at timing 2 has shortened the average stipe length by 1.4 cm, resulting in a significantly higher PD/SL ratio than control. In the second harvest, biological and economic yield increased by 22.0% and 25.6% in C1t1, 28.0% and 25.1% in C2t2, and 25.8% and 25.9% in C2t3.

**Table 4:** Effect of nano-amino application on the second harvest

Indicators	Treatments							
	C0t0	C1t1	C1t2	C2t1	C2t2	C1t3	C2t3	*P*_*value*_
Bunches number	3.3ab	3.3ab	5.3b	2.7a	3.3ab	3.3ab	5.0ab	ns
Fruit body number	9.0a	11.3ab	11.7ab	11.0ab	20.3c	9.0a	15.3b	0.001
Bunches weight (g)	74.8b	89.6b	45.0a	91.6b	133.3c	80.4b	84.4b	0.000
Fruit body weight (g)	19.5ab	23.6b	16.9a	17.3a	15.2a	30.0c	21.0ab	0.001
Pileus diameter (cm)	7.8bc	9.6d	7.5abc	7.3ab	6.7a	10.0d	8.4c	0.000
Stipe diameter (cm)	1.4ab	1.6b	1.7b	1.5ab	1.1a	1.7b	1.5ab	ns
Stipe length (cm)	5.3b	6.1b	3.9a	6.2b	6.3b	6.3b	5.5b	0.003
Pileus length (cm)	7.1b	8.0c	5.7a	6.7b	5.8a	7.9c	6.9b	0.000
PD/SL	1.4bc	1.6c	1.9d	1.2ab	1.1a	1.6cd	1.6cd	0.002
Biological yield (g/bag)	233.2a	298.8b	251.8a	230.1a	324.0b	264.2a	314.5b	0.000
Economic yield (g/bag)	201.4ab	270.9c	212.7ab	181.5a	268.8c	228.3b	271.7c	0.000

Values are means; means within the same row followed by the same letters are not significantly different at *P* < 0.05 according to Duncan’s multiple range test, ns: non-significant effect

### Mushroom production at the third harvest

Supplementation of the growing substrate at spawning with 3 g/kg nano-amino caused a significant increase in bunches number (by 3.3 on average), and fruit body number (by 11.7 on average), coupled with a significant reduction in bunches weight (by 25.6 g on average), and fruit body weight (by 26.1 g on average) at the third harvest compared with control (Table 5). Otherwise, when the same product dose was applied twice (treatment C1t3), it improved bunches weight (by 82.4 g on average) and fruit body weight (by 45.5 g on average). In all treatments, the pileus diameter and stipe diameter were significantly lower at the third harvest compared to control. A similar trend was observed for stipe length (except in C1t3), pileus length (except in C1t3), and PD/SL ratio (except in C2t3). Production at the third harvest was comparable to control, except in C1t3, where biological and economic yields increased by around 36.7% and 36.4%.

**Table 5:** Effect of nano-amino application on the third harvest

Indicators	Treatments						
	C0t0	C1t1	C2t1	C2t2	C1t3	C2t3	*P*_*value*_
Bunches number	3.0a	6.3b	3.0a	4.7ab	2.3a	4.7ab	0.018
Fruit body number	6.3a	18.0c	10.7b	11.0b	6.7a	7.0a	0.000
Bunches weight (g)	51.2b	25.6a	43.2ab	50.4b	133.6c	34.0ab	0.000
Fruit body weight (g)	33.4b	7.3a	10.2a	11.3a	78.9c	15.4a	0.000
Pileus diameter (cm)	12.2d	5.8a	7.1b	6.6ab	9.4c	9.1c	0.000
Stipe diameter (cm)	1.5e	0.4a	1.0bc	0.8b	1.3d	1.1c	0.000
Stipe length (cm)	5.1b	3.7a	3.9a	4.2a	5.8c	4.0a	0.000
Pileus length (cm)	8.5c	5.8a	5.9a	6.1a	8.0c	7.0b	0.000
PD/SL	2.4b	1.6a	1.8a	1.6a	1.6a	2.3b	0.001
Biological yield (g/bag)	136.8ab	164.5b	120.6a	145.0ab	216.3c	149.7b	0.000
Economic yield (g/bag)	115.2ab	131.9b	98.9a	126.6ab	181.1c	119.9ab	0.000

Values are means; means within the same row followed by the same letters are not significantly different at *P* < 0.05 according to Duncan’s multiple range test, ns: non-significant effect

### Total production per treatment

The harvest’s number (Table 6) rose from 3.0 in control to 4.0 in C1t3 and decreased to 2.0 in C1t2. Additionally; the double application of 3 g/kg nano-amino improved the total biological and economic yields by around 23.2 and 25.3%, respectively, and resulted in the highest biological efficiency (117.3%).

**Table 6:** Effect of nano-amino application on total production

Indicators	Treatments							
	C0t0	C1t1	C1t2	C2t1	C2t2	C1t3	C2t3	*P*_*value*_
Number of harvests	3.0b	3.0b	2.0a	3.3bc	3.3bc	4.0c	3.3bc	0.001
Biological efficiency (%)	90.0cd	98.1d	54.4a	78.8b	84.8bc	117.3e	89.1bcd	0.000
Total biological yield (g/bag)	799.5cd	871.6d	482.9a	699.9b	753.5bc	1041.9e	791.2bcd	0.000
Total economic yield (g/bag)	691.5c	728.0c	396.5a	590.0b	643.4bc	925.4d	687.6c	0.000

Values are means; means within the same row followed by the same letters are not significantly different at *P* < 0.05 according to Duncan’s multiple range test, ns: non-significant effect

### Variation of biological yield per harvest and in total

Results in Table 7 demonstrated that the total biological yield was strongly positively correlated with biological yields of the first (*R* = 0.51, *P* = 0.02) and third (*R* = 0.91, *P* = 0.00) harvests, but was the most strongly correlated with the latter. The most significant model (model1, *R*^2^ = 0.84) resulting from stepwise regression (Table 8) showed that the biological yield of the third harvest had a higher positive contribution (highest positive coefficient) to variation in total biological yield compared to that obtained at the first harvest.

**Table 7:** Pearson’s correlations between mushroom indicators and biological yield

Independent	BY(H1 )		Independent	BY(H2)		Independent	BY(H3)	
	*R*	*P*_*value*_		*R*	*P*_*value*_		*R*	*P*_*value*_
BN (H1)	0.54	0.07	BN (H2)	0.29	0.10	BN (H3)	0.01	0.48
FBN (H1)	0.95	0.00*	FBN (H2)	0.72	0.00*	FBN (H3)	0.00	0.50
BW (H1)	0.47	0.10	BW (H2)	0.51	0.01*	BW (H3)	0.72	0.00*
FBW (H1)	− 0.29	0.23	FBW (H2)	0.02	0.46	FBW (H3)	0.75	0.00*
PD (H1)	− 0.43	0.12	PD (H2)	0.09	0.34	PD (H3)	0.05	0.42
SD (H1)	− 0.83	0.00*	SD (H2)	− 0.27	0.12	SD (H3)	0.02	0.47
SL (H1)	0.27	0.24	SL (H2)	0.31	0.08	SL (H3)	0.64	0.00*
PL (H1)	− 0.69	0.02*	PL (H2)	− 0.04	0.43	PL (H3)	0.32	0.10
BYT	0.51	0.02*		0.10	ns		0.91	0.00*

H: harvest, BY: biological yield, BN: bunches number, FBN: fruit body number, BW: bunches weight, FBW: fruit body weight, PD: pileus diameter, SD: stipe diameter, SL: stipe length, PL: pileus length, BYT: total biological yield

* Significant correlation at the 0.05 level (1-tailed)

**Table 8:** Stepwise regression models showing the relation between the biological yield (per harvest/in total) and mushroom characteristics

Dependent	Predictors	Model equation	Adjusted *R*^*2*^	Model n
BYT	BY(H1), BY(H2), BY(H3)	TBY = 0.76 × BY(H1) – 0.54 × BY(H2) + 3.062 × BY(H3) + 187.047	0.84	Model 1
BY(H1)	FBN(H1)	BY = 8.65 × FBN + 261.85	0.89	Model 2
	FBN(H1), SD (H1)	BY = 6.5 × FBN – 82.21 × SD + 451.774	0.98	Model 3
BY(H2)	FBN(H2)	BY = 6.7 × FBN + 189.97	0.50	Model 4
	FBN(H2), PD (H2)	BY = 9.68 × FBN + 20.01 × PD – 11.24	0.80	Model 5
BY(H3)	FBW(H3)	BY = 0.95 × FBW + 130.76	0.53	Model 6
	FBW(H3), BN (H3)	BY = 1.39 × FBW + 12.06 × BN + 70.84	0.81	Model 7
	FBW(H3), BN (H3), SD(H3)	BY = 1.54 × FBW + 8.63 × BN – 34.66 × SD + 115.80	0.88	Model 8

BYT: total biological yield, H: harvest, BY: biological yield, FBN: fruit body number, SD: stipe diameter, PD: pileus diameter, FBW: fruit body weight, BN: bunches number.

The biological yield of the first harvest was positively correlated with the fruit body number (*R* = 0.95, *P* = 0.00), and negatively correlated with stipe diameter (*R* = – 0.83, *P* = 0.00) and pileus length (*R* = – 0.69, *P* = 0.02), obtained at the same harvest. The most significant model resulting from stepwise regression (model 3, *R*^2^ = 0.98) showed that the stipe diameter was the predictor contributing the most (highest coefficient) to variation in the first harvest’s biological yield. Moreover, there was a strong positive correlation between the biological yield and fruit body number (*R* = 0.72, *P* = 0.00), and bunches weight (*R* = 0.51, *P* = 0.01) of the second harvest. The fruit body number was a good predictor of the variation in biological yield of the second harvest (positive coefficient, model 4); however, the most significant model of stepwise regression (model 5, *R*^2^ = 0.80) depicted a stronger contribution of the pileus diameter (higher coefficient). Biological yield of the third harvest was strongly and positively correlated with bunches weight (*R* = 0.72, *P* = 0.00), fruit body weight (*R* = 0.75, *P* = 0.00) and stipe length (*R* = 0.64, *P* = 0.00). Besides, fruit body weight, bunches number, and stipe diameter were good predictors to variation of the biological yield of the third harvest. However, the stipe diameter had the highest contribution (highest coefficient, model 8). Noteworthy is that stipe diameter had a negative contribution to biological yields of the first and third harvests.

### Fiber fractions in the residual substrate

Analysis of the spent substrate generated from each treatment (Table 9) showed that residual cellulose, hemicellulose, NDF, and ADF were the lowest in the control substrate than substrates initially treated by nano-amino. Concerning the latter, residual cellulose was significantly reduced in C2t3 compared to the remaining substrates (reduction by 9.4 units compared to the initial substrate). It was also more significantly reduced in C2t1 and C2t3 than in C1t1 and C1t3, and in C1t2 than in C2t2. Hemicellulose was more degraded with 5 g/kg than 3 g/kg nano-amino at all tested timings (reduction range of 8.5–11.6 units compared to 0.1–6.4 units, respectively). As a result, residual NDF was higher in the later substrates. Residual ADF was significantly lower in substrates initially treated with 5 g/kg at spawning than 3 g/kg. However, opposite results were obtained when the same respective doses were initially applied after the first harvest. Lignin was the more degraded when initial substrates were treated by the lower dose compared to the highest (reduction range of 3.03–5.81 units compared to 1.40–1.69, respectively), and was the most pronounced in C1t3 substrates. In the same substrates, residual ADL was the most reduced in comparison with the control.

**Table 9:** Fiber fractions analysis (% dry weight) of initial and residual substrate (s)

Fiber fractions	Initial	Residual							
		C0t0	C1t1	C2t1	C1t2	C2t2	C1t3	C2t3	*P*_*value*_
Cellulose	35.95	23.33a	33.15 g	29.50e	27.85c	28.83d	30.70f	26.54b	0.000
Hemicellulose	13.14	0.98a	8.74f	1.52b	13.03g	3.11c	6.73e	4.59d	0.000
Lignin	6.59	4.46d	2.82b	5.14f	3.56c	5.19f	0.78a	4.90e	0.000
NDF	59.26	32.51a	49.84e	40.69b	50.14e	45.23d	43.55c	41.12b	0.000
ADF	46.11	31.52a	41.09d	39.17c	37.11b	42.12e	36.83b	36.52b	0.000
ADL	10.17	8.20b	7.95b	9.67cd	9.26c	9.68cd	6.12a	9.98d	0.000

Values are means; means within the same row followed by the same letters are not significantly different at *P* < 0.05 according to Duncan’s multiple range test, NDF: neutral detergent fiber, ADF: acid detergent fiber, ADL: acid detergent lignin

### Protein and amino acid content in mushrooms

Mushroom protein content (Table 10) decreased due to the nano-amino application compared to control, except in C2t1. There was an in some essential amino acids in C1t1 (threonine, valine, isoleucine, leucine, and histidine) and C2t3 (Threonine, histidine, and methionine) mushrooms. Also, nano-amino treatment had a significant effect on histidine content, which was improved in all treatments compared with control. On the contrary, it caused a decrease in the mushroom’s arginine content. Alanine content was higher than control, except in C1t3 and C2t3. Glutamic acid increased by 0.48, 0.28, 0.20, and 1.13 units in C1t1, C2t2, C1t3, and C2t3, respectively. Proline content was higher by 0.16 and 0.14 units in C1t1 and C2t3 than in control.

**Table 10:** Protein content and amino acid profile of mushrooms (dry basis, % of total amino acids)

	C0t0	C1t1	C1t2	C2t1	C2t2	C1t3	C2t3	*P*_*value*_
Essential amino acids								
Threonine	0.96b	1.08d	0.93a	0.94ab	1.03c	0.96b	1.04c	0.00
Valine	0.86bc	0.91d	0.76a	0.83b	0.87c	0.74a	0.75a	0.00
Isoleucine	0.66cd	0.69e	0.58a	0.63bc	0.66d	0.57a	0.61b	0.00
Leucine	1.11c	1.19e	1.01a	1.00a	1.14d	1.04b	1.06b	0.00
Phenylalanine	0.68ab	0.76b	0.66a	0.69ab	0.72ab	0.71ab	0.68a	ns
Histidine	2.77a	4.22f	4.35g	3.93c	4.05d	3.65b	4.19e	0.00
Lysine	0.91bc	0.94c	0.87ab	0.84a	0.90bc	0.89b	0.92bc	0.00
Methionine	0.27a	0.29a	0.26a	0.30ab	0.28a	0.28a	0.33b	0.00
Non-essential amino acids								
Aspartic acid	1.70a	1.98d	1.71a	1.79b	1.90c	1.77ab	2.05d	0.00
Serine	0.85a	1.06c	0.95abc	0.90ab	1.02bc	0.96abc	1.02bc	ns
Glutamic acid	4.21c	4.69e	3.51a	3.95b	4.49d	4.41d	5.34f	0.00
Proline	0.66ab	0.82c	0.65a	0.70abc	0.78bc	0.66ab	0.80c	ns
Glycine	0.82ab	0.90c	0.77a	0.78a	0.86ab	0.76a	0.83ab	0.00
Alanine	1.69b	2.19f	1.92d	1.81c	2.10e	1.40a	1.72b	0.00
Arginine	1.59e	1.48c	1.47c	1.53d	1.42b	1.29a	1.39b	0.00
Cystine + cysteine	0.15a	0.25c	0.24c	0.15a	0.24c	0.18b	0.17b	0.00
Protein (% dry weight)								
Protein	2.92d	2.95d	2.82c	3.25a	2.23a	2.24a	2.64b	0.00

Values are means; means within the same row followed by the same letters are not significantly different at *P* < 0.05 according to Duncan’s multiple range test, ns: non-significant difference

## Discussion

Oyster mushroom develops well and gives best yield at pH slightly basic in nature (Khan et al. 2013). The application of a low dose of nano-amino, with high amounts of CaCO_3_ and CaO, from the spawning time, may have affected the initially low substrate pH (5.2) at the early growth stages of mycelium, thus the harvest date and the biological efficiency in treated substrates. However, the product’s effect on the substrate pH, consequently, on the growth and production of the mushroom requires further investigation in future studies to prove the above-stated assumptions.

Although biological yield was reduced at the first harvest, mostly due to reduced fruit body numbers, heavier mushrooms were obtained with a longer and a thicker stipe. Supplementation at spawning also had a delayed positive effect on the number of bunches and fruit bodies produced at the third harvest.

After testing the effect of supplementation after the first harvest, it seemed that a high product dose was essential to influence substrate productivity directly. In point of fact, treating the growing substrate with 5 g/kg at this timing has optimized the biological yield obtained at the second harvest (improvement of 28.0% compared to control). On the contrary, the application of 3 g/kg at this timing has limited the production to only two flushes. These findings suggest the slow release of nutrients by the tested product into the growing substrate. Due to their extensive surface area, nanoparticles can hold an abundance of nutrients and release it slowly and steadily facilitate their uptake (Selva and Balakrishnan 2017). Applying the product twice with 3 g/kg was the most effective; not only had it increased the number of harvests to four, but it optimized as well the biological efficiency (117.3%) from the first three harvests. A biological efficiency between 50 and 63% was obtained using wheat straw and spent Pleurotus substrate as a base material supplemented with a 120 g/6 kg dose of a protein-rich additive Calprozime^®^ (Picornell-Buendía et al. 2015). Different tests to investigate the practicality of reusing such substrate in new production cycles had found, because of supplementation, increments of biological efficiency somewhere in the range of 51 and 70% (Zied et al. 2011). Nitrogen can be transported into the fungus’s living cell in the form of amino acids (Mikeš et al. 1994). The product applied was rich in amino acids, essential during the mycelial maturation stage (Du et al. 2019). When added to the growing substrate, amino acids, as source of nutrients, could be more easily assimilated than proteins present in the initial substrate. Mycelia development was ameliorated due to the addition of the amino acids glycine and leucine to the growing culture medium (Adebayo-Tayo et al. 2011).

*Pleurotus ostreatus* can decompose the cellular wall components present in the raw lignocellulosic material, like cellulose, lignin, and hemicellulose through the action of complex oxidative and hydrolytic enzymatic systems (Castro 2003; Fernández-Fueyo et al. 2016). Among others, hemicellulases, cellulases, and ligninases enzymes degrade long and insoluble parts of lignocellulosic materials into soluble components of low molecular weight that are taken by intracellular enzymes of fungi for their nutrition (Kurt and Buyukalaca 2010; Picornell-Buendía et al. 2015). Hemicellulose, cellulose, and lignin serve as an energy source for fungal growth because they contain carbon, hydrogen, and oxygen, clarifying their decrease along the cultivation cycle (Andrade et al. 2010). Lignin probably acts as a barrier to prohibit the mushroom from attacking polysaccharides. Therefore, access to holocellulose, the carbon and the energy source for this species, is enabled after lignin degradation (Xiao et al. 2017). *P. ostreatus* secretes Manganese peroxidase (MnP), one major oxidative enzyme responsible for lignin oxidation (Wan and Li 2012). Therefore, the highest lignin degradation obtained following the double application of 3 g/kg of Lithovit^®^-Amino25 containing manganese may be associated with an improved *P. ostreatus* MnP activity. Improvement in lignin degradation may be the cause behind the highest biological efficiency obtained in such treatment. This assumption may be confirmed by further investigating the extracellular enzymes’ secretion by a nano-amino supplemented mycelium.

In general, the abundance of nano-amino in the growing substrate significantly affected the pileus length of harvested mushrooms. In the treatment C1t3 the weight of fruit bodies increased at the third harvest, mainly because of larger pileus (length and diameter). Picornell-Buendía et al. (2015) obtained fruiting bodies of excellent weight on spent mushroom substrate supplemented with protein additives. Also, the mushroom shape was more uniform among consecutive harvests (almost similar PD/SL ratio at the second and third harvests). Still, mushrooms produced in such treatment at the second harvest were more marketable than those obtained in the non-treated substrate (higher PD/SL ratio). Mushrooms having large pileus and short stipes are more acceptable at the market (Synytsya et al. 2008).

The mushroom nutrient composition is affected by the substrate composition and properties (El Sebaaly et al. 2018, 2019; Abou Fayssal et al. 2020). The mushroom nutritional value may be improved as by applying nano-amino at spawning; proteins increase with the highest dose used, and essential amino acids with the lowest one. A similar improvement in mushroom protein content was reported on substrates based on wheat straw and SOS, enriched by protein-rich additives, wheat bran, or commercial additives (Picornell-Buendía et al. 2016a, b).

Pieces of evidence were provided by the present study that Lithovit^®^-Amino25 can be used in *P. ostreatus* cultivation, especially with 3 g/kg applied twice during the production cycle. In addition to the improvement in biological yield, farmers may optimize their benefits from *P. ostreatus* by saving in the initial substrate cost and the cost of supplements, since lower product doses are required to obtain good results.

## Data Availability

Authors apologize for not being able to share the data because it belongs to a PhD study that is not yet defended.

## Abbreviations

C: Concentration
t: Timing
NDF: Neutral detergent fiber
ADF: Acid detergent fiber
*P. ostreatus*: *Pleurotus ostreatus*
SMS: Spent mushroom substrate
CaCO_3_: Calcium carbonate
CaO: Calcium oxide
SiO_2_: Silicon dioxide
N: Nitrogen
MgO: Magnesium oxide
Fe: Iron
Mn: Manganese
L: Lithovit
SOS: Spent oyster substrate
C/N: Carbon/nitrogen ratio
AOAC: Association of Official Analytical Chemists
LFDCA: Lebanese Food Drugs and Chemicals Administration
ADL: Acid detergent lignin
OPA: o-Phthalaldehyde
DAS: Days after spawning
BE: Biological efficiency
SPSS: Statistical Package for Social Sciences
ANOVA: Analysis of variance
PD/SL: Pileus diameter/stipe length ratio
BY: Biological yield
BN: Bunches number
FBN: Fruit body number
FBW: Fruit body weight
PD: Pileus diameter
SD: Stipe diameter
SL: Stipe length
PL: Pileus length
BYT: Total biological yield
H: Harvest
